# The effect of bright light therapy on glycemic control and cortisol rhythmicity in depression: a randomized controlled trial

**DOI:** 10.3389/fpsyt.2026.1743465

**Published:** 2026-02-11

**Authors:** Jie Fang, Xinyu Li, Shukun Zhu, Qinghong Hu, Ting Wang, Jiakuai Yu, Ximing Qin, Daomin Zhu

**Affiliations:** 1 The School of Mental Health and Psychological Sciences, Anhui Medical University, Hefei, China; 2 Department of Sleep Medicine, Affiliated Psychological Hospital of Anhui Medical University, Hefei, China; 3 Department of Sleep Medicine, Anhui Mental Health Center, Hefei, China; 4 Department of Sleep Medicine, Hefei Fourth People's Hospital, Hefei, China; 5 Institutes of Physical Science and Information Technology, Anhui University, Hefei, China

**Keywords:** bright light therapy, cortisol, depression, fasting blood glucose, mesor, metabolic disturbances

## Abstract

**Background:**

Depressed patients with comorbid metabolic disorders have poorer quality of life and prognosis. Pharmacological interventions carries risks of liver and kidney toxicity, which highlights the need for safer non-pharmacological alternatives. Experimental data suggest that light exposure modulates cortisol secretion, thereby influencing metabolic outcomes in depression. We hypothesized that bright light therapy may ameliorate metabolic disturbances by modulating cortisol secretion.

**Methods:**

In this randomized controlled trial, hospitalized patients with depression were assigned to receive either bright light therapy (BLT) or dim-light control condition. The pre−specified primary endpoint was the change in fasting blood glucose (FBG) and cortisol rhythm indices from baseline to post−treatment. Secondary endpoints included changes in other glycolipid parameters, and scores on the Hamilton Depression (HAMD) and Anxiety (HAMA) scales. Treatment effects were evaluated using linear mixed−effects models with baseline adjustment.

**Results:**

After the 2−week intervention, the BLT group showed a significant reduction in fasting blood glucose (95% CI: 0.280 to 0.600; *p* < 0.001) and cortisol mesor (95% CI: −2.677 to −0.064; *p* = 0.040) compared to the control group. Within the BLT group, the change in FBG was positively associated with the change in cortisol mesor after adjusting for covariates (β = 0.053, 95% CI: 0.016 to 0.122, *p* = 0.036).

**Conclusions:**

The findings of this study support a potential mechanism whereby BLT modulates cortisol rhythmicity, which in turn may contribute to improved glycemic control, pointing to its potential therapeutic benefit for addressing metabolic disturbances in depression.

**Clinical Trial Registration:**

https://www.chictr.org.cn/bin/project/edit?pid=260569, identifier ChiCTR2500097364.

## 1 Introduction

Depression has become a significant cause of disability worldwide, adversely impacting both psychological well-being and physical health. Epidemiological data reveal an 88.52% surge in prevalence rates from 1990 to present, accompanied by a substantial rise in its disease burden worldwide (1, 2). Metabolic disturbances are another major health threat (3), epidemiologic evidence consistently indicates a comorbidity between depression and metabolic disturbances (4), with studies reporting a significantly higher prevalence of metabolic disturbances among depressed individuals compared to the controls (5). Patients with depression often exhibit various disease-specific physiological dysfunctions, including characteristic symptoms such as unbalanced dietary patterns, sleep disturbances, and significantly reduced physical activity levels (6). Certain behaviors associated with depression are established risk factors for Metabolic disturbances onset. Furthermore, standard pharmacological treatments for depressive disorders may directly influence multiple metabolic disturbances, potentially contributing to the identified clinical associations (7). It has been suggested that the metabolic disturbances (8), especially glycemic abnormalities, responds poorly to antidepressants, leading to a more chronic course in depressed patients (9, 10). Therefore, a non-pharmacological treatment is necessary.

Cortisol, a steroid hormone belonging to the glucocorticoid class, is primarily produced within the adrenal cortex’s zona fasciculata. The synthesis and release of this Hormones are precisely controlled by the hypothalamic–pituitary–adrenal (HPA) axis. Additionally, the secretion of cortisol follows a circadian pattern that aligns with the external alternation of light and darkness (11). Moreover, changes in cortisol levels are contingent upon the functional integrity of the suprachiasmatic nucleus (SCN) (12). Compelling evidence indicates that dysregulation of the HPA axis plays a significant role in the pathophysiology of depression during adulthood (13, 14). Cortisol secretion is altered in up to 80% of depressed patients (15), and cortisol levels are increased in depressed patients (16). There is evidence of a link between hypercortisolism and glycemic abnormalities (17). Increased cortisol secretion, reflecting potential HPA axis dysfunction, can contribute to elevated blood glucose levels (18).

Light is the most potent zeitgeber and a critical regulator of circadian rhythms (19). Light therapy, a non-pharmacological intervention known for its excellent safety record, has attracted increasing attention in recent years owing to its promising role in managing depressive disorders. As a complementary approach to pharmacotherapy for depression, a substantial body of research has confirmed its favorable efficacy and safety profile (20, 21). The underlying mechanism of this therapy is likely associated with its influence on circadian rhythm modulation, mediated via the SCN, which serves as the central pacemaker for circadian cycles in mammals. The SCN serves as a central regulator of multiple physiological functions, notably governing circadian rhythms such as sleep-wake patterns, thermoregulation, cardiovascular homeostasis, and neuroendocrine activity (including the release of cortisol) (22). By modulating SCN function, light therapy can effectively stabilize circadian rhythms, thereby alleviating depressive symptoms (23). Interestingly, growing evidence suggests an important link between circadian rhythm disruption and metabolic disturbances in humans (24–26). This connection is further supported by case reports documenting light therapy’s positive effects on glucose metabolism (27, 28) highlighting its potential dual role in both mood regulation and metabolic improvement, particularly in patients with mood disorders. Notably, one potential mechanism could be the role of light therapy in restoring normal cortisol regulation, given empirical evidence indicating its efficacy in attenuating hypercortisolemia in individuals with depression (29). These findings collectively suggest light therapy may offer a multifaceted therapeutic approach by simultaneously addressing mood disturbances and metabolic disturbances associated with circadian rhythm disruption.

Based on the rationale that light therapy shows promise in alleviating depressive symptoms but its effects on glucose and lipid metabolism and underlying physiological pathways remain unclear, we conducted a randomized controlled trial to test the following primary hypotheses: (H1) compared to dim light therapy, bright light therapy (BLT) will reduce fasting blood glucose levels in individuals with depression; (H2) BLT will reduce the mesor and amplitude of the circadian cortisol rhythm; and (H3) within the BLT group, changes in cortisol parameters will correlate with changes in fasting blood glucose and depressive symptoms.

## 2 Methods

### 2.1 Trial design

Eligible subjects enrolled in this randomized controlled trial were distributed, using a randomized approach, into two parallel groups: a bright light therapy intervention or a control condition. The participants were unaware of the grouping. The Psychological Hospital of Anhui Medical University Institutional Review Board (HFSY-IRB-YJ-KYXM-ZDM) approved of the study protocol. Upon enrollment, all volunteers provided their informed consent in writing after they received a complete overview of the experimental protocols and goals. Prior to commencement, this study was registered at the Chinese Clinical Trial Registry and assigned the trial ID ChiCTR2500097364.

### 2.2 Participants and enrollment criteria

The study cohort comprised 61 inpatients diagnosed with depression according to DSM-IV criteria. Participants were recruited from the Sleep Disorders Unit of the Psychological Hospital at Anhui Medical University between February and July 2025. Enrollment was strictly contingent upon meeting all predefined inclusion criteria: 1) aged between 18 and 65; 2) diagnosed with depression according to DSM-IV criteria; 3) graduated from elementary school or above; and 4) following a comprehensive explanation of the research protocol, written informed consent was obtained from all participants. Exclusion criteria encompassed: 1) a history of intellectual disability, organic brain injury, or other primary psychiatric conditions; 2) receipt of electroconvulsive therapy or transcranial magnetic stimulation within the three months prior to enrollment; 3) the presence of diagnosed systemic or neurological diseases—such as diabetes mellitus, thyroid disorders, hypertension, cardiac diseases, narrow-angle glaucoma, epilepsy, or dementia; 4) pregnancy (current, planned, or during breastfeeding); and 5) any ophthalmic pathology contraindicating phototherapy, such as cataracts, macular degeneration, glaucoma, retinal lesions, or blindness; 6)current smoking, drinking caffeinated beverages, using blood sugar-lowering or lipid-regulating drugs, and using drugs such as hydrocortisone or prednisone were not included in this research. However, former smokers were eligible if they had abstained for at least 6 months prior to enrollment. During the study, other medications were allowed.

### 2.3 Randomization, concealment and blinding

Randomization of participants to the bright light therapy versus the control group was performed via a centralized web-based platform. A computer-generated sequence was used to assign participants to their respective groups. To ensure concealment, this sequence was kept confidential until the interventions were administered, the participants remained unaware of their group assignment throughout the trial. Furthermore, An unblinded researcher was assigned tasks to administer and assign throughout the experiment, and all observers who scored behavior and performed data analysis were blinded to treatment assignment.

### 2.4 The intervention

#### 2.4.1 Environmental light control and preparation

Prior to and throughout the intervention period, all participants adhered to a fixed sleep–wake schedule within the hospital ward. Ambient lighting was strictly controlled: illumination was maintained at approximately 400 lux from 06:00 to 22:00 and reduced to ≤ 20 lux from 22:00 to 06:00. Participants were admitted to the ward one day before baseline assessments to acclimatize and to avoid prolonged exposure to uncontrolled natural light.

#### 2.4.2 Light therapy intervention

This intervention measure involves daily morning phototherapy for 14 days. A full-spectrum white light box (size: 25 × 50 cm; wavelengths > 450 nanometers of blue light are not filtered, color temperature: 6000K) is placed 50 cm above the participants’ heads at an angle. The illuminance at the eye level is measured using a photometer to calibrate the light intensity. Participants in the BLT group receive 10,000 lux of light, while those in the control group receive 100 lux of weak light. Each treatment lasts for 30 minutes and is conducted between 9 and 10 in the morning.

#### 2.4.3 Saliva sampling

Trained nurses collected saliva samples from patients. On two consecutive days, saliva was collected at 8:00, 11:00, 14:00, 19:00, 20:00, 21:00, 22:00, 01:00, and 05:00. Saliva samples (≥1 ml) were collected using specialized tubes (Salivette^®^, Sarstedt AG & Co, Nümbrecht, Germany) and preserved at -40 °C pending subsequent analysis. Participants were instructed to refrain from consuming caffeinated or alcoholic beverages throughout the sampling period. They were also advised to avoid tooth brushing or gum chewing for at least 15 minutes prior to each sample collection. Trained experimenters collected samples at night at the dimmest possible light degrees.

### 2.5 Assessment

#### 2.5.1 Glucose and lipid metabolism indexes

Following an overnight fast, 5 mL of peripheral venous blood was collected both before and upon completion of the two-week light therapy intervention. Subsequent analysis of glucose and lipid metabolism indicators was performed by the hospital’s clinical laboratory.

#### 2.5.2 Salivary cortisol measurement

Salivary cortisol concentrations were determined using a commercial enzyme-linked immunosorbent assay (ELISA) kit (DRG International, Inc.) in strict accordance with the supplier’s instructions. The detection limit of the assay was 0.09 ng/mL. The intra-assay coefficient of variation (CV) was measured at 6.1% for a concentration of 3.08 ng/mL and decreased to 2.6% at 20.14 ng/mL. For inter-assay precision, the CV values were 13.6% at 0.64 ng/mL and 4.3% at 19.78 ng/mL.

#### 2.5.3 Clinical symptoms

The Hamilton Anxiety Scale (HAMA) and Hamilton Depression Inventory (HAMD) are established tools for gauging anxiety and depression severity, respectively. The former contains 14 items, while the latter comprises 24 (30). The HAMA and HAMD scales, which range from 0 to 56, respectively, share a common scoring system where elevated scores correspond to a greater severity of symptomatology. In the HAMD, the total score for no depression is 0 ~ 8; mild to moderate depression is 21 ~ 35; and major depression is ≥ 36.

### 2.6 Side effects and adverse reactions

The assessment of possible adverse reactions and side effects after two weeks of light intervention was done and recorded by the psychiatrist (31).

### 2.7 Sample size

Utilizing G*Power 3.1, a minimum sample size was calculated based on a prior effect size estimate (Cohen’s d = 0.44) derived from a randomized controlled trial investigating light therapy in patients with type 2 diabetes and comorbid depression (32). The effect size corresponds to the between-group difference in fasting blood glucose observed at the end of the intervention period in that study. This calculation employed a two-tailed test with an alpha (α) level of 0.05 and was set to achieve a statistical power of 80%. The analysis indicated that 21 participants per group (total N = 42) were required. Accounting for an estimated 30% dropout rate (e.g., attrition, missing data), we planned to recruit 61 participants to ensure sufficient statistical power in the final analysis.

### 2.8 Statistical analysis

All analyses followed the intention-to-treat (ITT) principle. Baseline characteristics were compared using independent-samples t-tests (continuous variables) and chi-square tests (categorical variables), with data presented as mean ± standard. Based on the *a priori* hypothesis that BLT modulates cortisol secretion to improve metabolic dysregulation in depression, two primary analyses were pre-specified: (1) a linear mixed-effects model (LMM) to test the Group × Time interaction effect on FBG and cortisol mesor, and (2) within the BLT group, a multiple linear regression model examining the association between posttreatment changes in FBG and cortisol mesor, adjusted for age, sex, medication dosage (fluoxetine-equivalent), and illness duration. For all longitudinal outcomes, LMMs included a participant-specific random intercept and baseline covariates to estimate fixed effects for Group, Time, and their interaction. Secondary and exploratory analyses included LMMs for other metabolic and cortisol parameters, between-group comparisons of HAMD and HAMA scale scores, correlation analyses between cortisol and clinical changes, and sex-stratified sensitivity analyses.

We fitted a nonlinear model to the cortisol data from each participant using the function Y = mesor + amplitude × sin(2π × (X – acrophase)/24), where the wavelength (period) was constrained to 24 hours. All fittings were conducted with GraphPad Prism 8.0. The system derived essential circadian rhythm variables—namely the mesor, amplitude, acrophase, and the coefficient of determination (R²)—automatically through computation to quantify the precision of the cosine curve fitting. Based on the fitted model, individual salivary cortisol rhythm variables such as period, acrophase, amplitude, and mesor were subsequently extracted.

**Figure 1:**
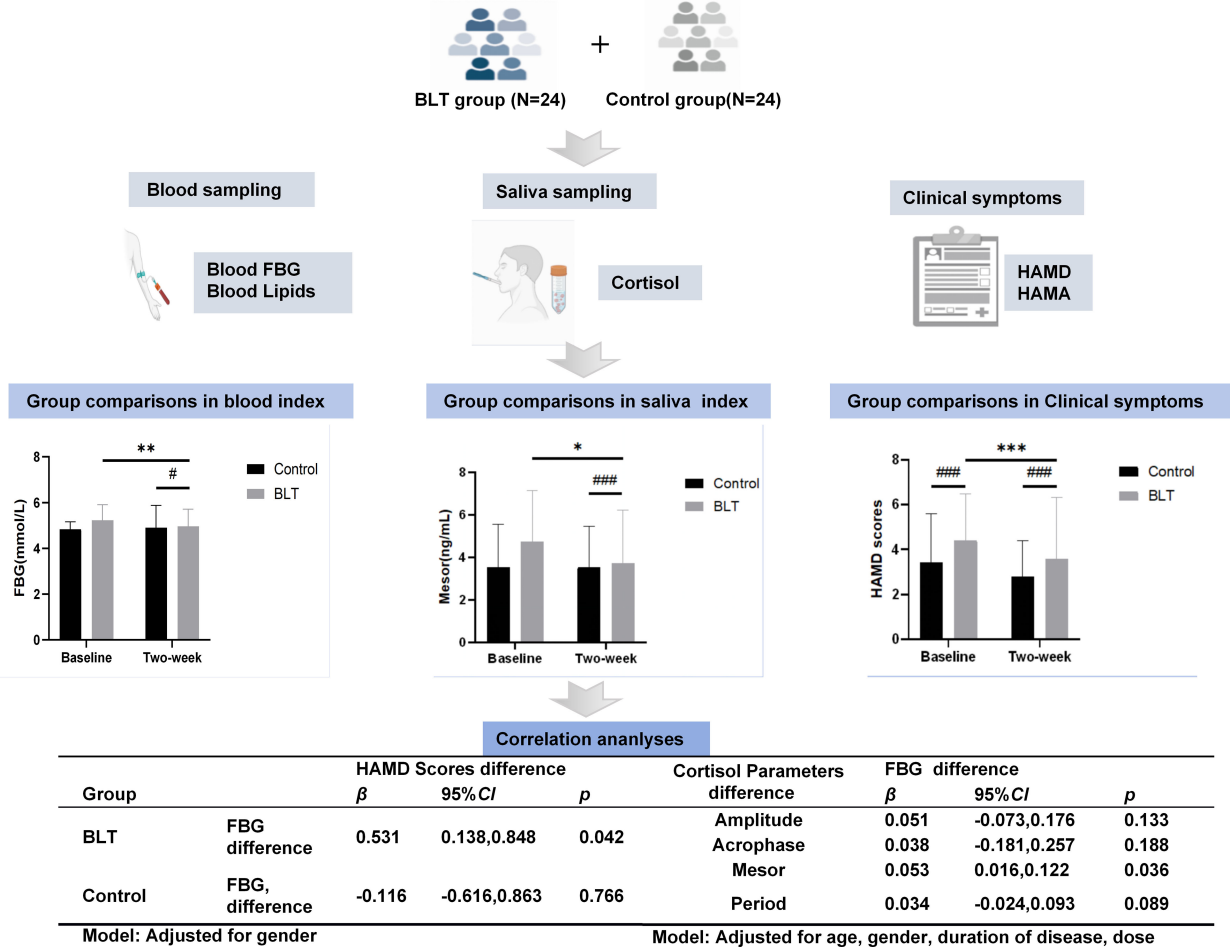
Study design and analysis pipeline. We collected the glycolipid metabolism indicators, salivary cortisol, and clinical scale data of 24 patients in the BLT group and 24 patients in the control group. We used group comparison and correlation analysis to explore the potential mechanism of bright light therapy in improving glucose and lipid metabolism in patients with depression. Abbreviations: BLT, bright light therapy; HAMA, Hamilton Anxiety Scale; HAMD, Hamilton Depression Scale; FBG, fasting blood FBG.

We utilized SPSS (version 25; IBM Corp) for all statistical computations and GraphPad Prism (version 8.0) for data visualization. Results were considered statistically significant if the p-value was below 0.05. A schematic diagram summarizing the study design and analysis pipeline is provided in **Figure 1**.

## 3 Results

### 3.1 The samples’ descriptive characteristics

**Figure 2:**
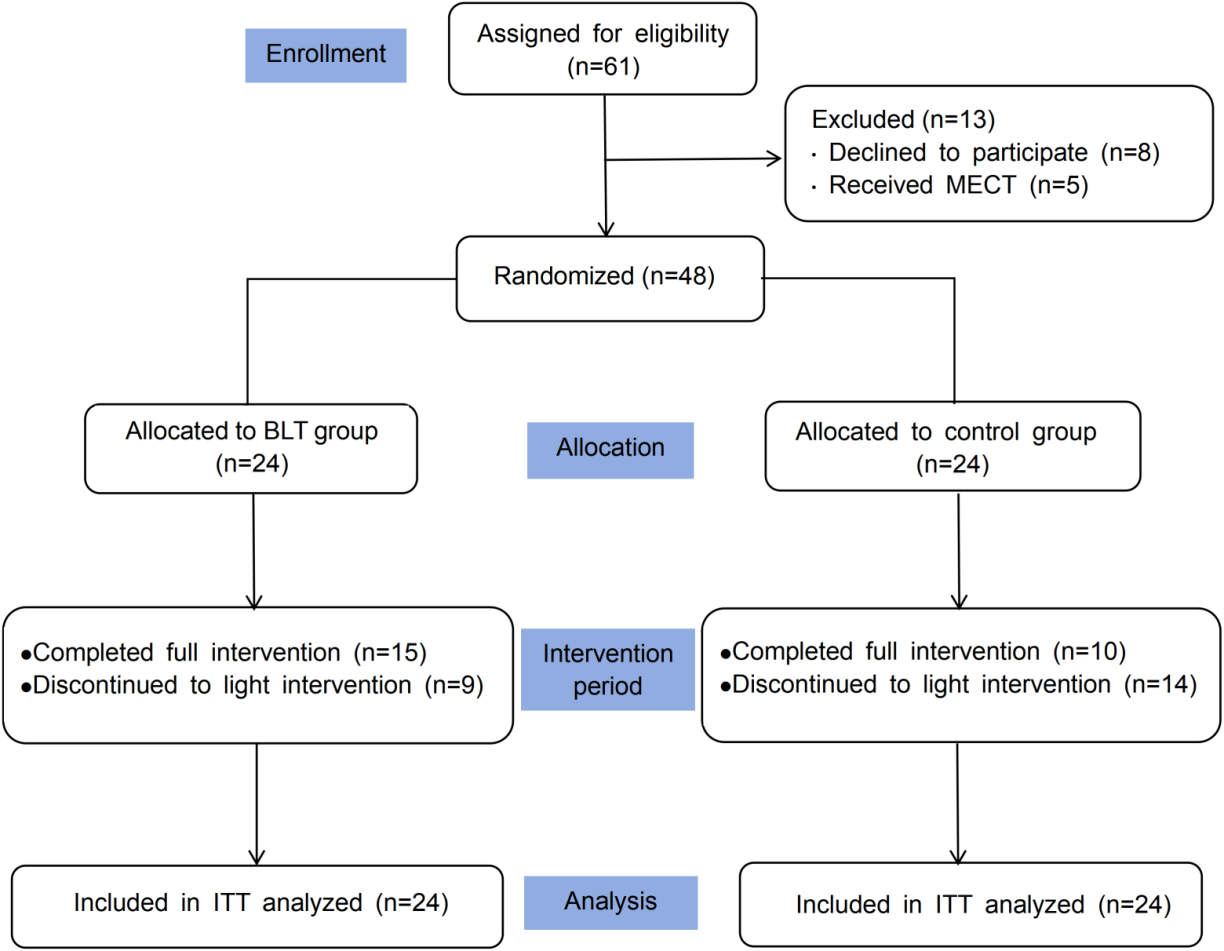
Study design diagram.

In this randomized controlled trial, a total of 48 individuals diagnosed with depression were assigned to either a bright light treatment group or a control group, as outlined in the participant flow diagram (**Figure 2**). Each group comprised 24 participants, with the control condition including 7 male and 17 female participants, while the bright light intervention group consisted of 14 males and 10 females. The average age of participants in the bright light group was 42.17 years (SD = 11.72), compared to 39.38 years (SD = 13.63) in the control condition. We found no significant differences in baseline demographic and clinical variables, such as age, education, illness duration, smoking status, or alcohol use, between the groups. However, a significant gender discrepancy was noted (*p* = 0.042). With respect to clinical features, both groups showed comparable scores on the HAMD and HAMA scales at baseline. And all enrolled patients scored ≥ 21 on the HAMD, indicating moderate to severe depression and confirming they were above the clinical cutoff. Furthermore, the categories and dosages of antidepressant medications used were similar across groups, with no significant variations (see **Table 1**).

**Table 1:** Demographic characteristics and clinical variables of the recruited subjects.

Variable	BLT group (n=24)	Control group (n=24)	*t/x ^2^*	*P*
Age, years, mean ± SD	42.17 ± 11.72	39.38 ± 13.63	-0.761	0.451
Gender, Female, n (%)	10 (42)	17 (71)	4.418	0.042
Duration of disease, mean ± SD	7.66 ± 8.31	4.68 ± 5.90	-1.403	0.168
Alcohol use (Yes), n (%)	8 (33)	5 (21)	-0.856	0.396
Smoking (Yes), n (%)	7 (29)	4 (17)	-1.020	0.313
Education, years, mean ± SD	11.17 ± 4.46	9.67 ± 4.66	-1.139	0.260
HAMD scores, mean ± SD	31.67 ± 12.11	30.78 ± 7.65	-0.292	0.772
HAMA scores, mean ± SD	19.86 ± 7.38	22.30 ± 6.83	1.142	0.260
Dose, mean ± SD	23.09 ± 13.30	27.15 ± 16.59	0.855	0.398
Antidepressant medications			0.602	0.550
SSRIs, n (%)	17 (71)	15 (62)		
SNRIs, n (%)	7(29)	9 (38)		

HAMD, Hamilton Depression Scale; HAMA, Hamilton Anxiety Scale; Dose, Equivalent dose of fluoxetine.

### 3.2 Adverse reactions

In the BLT group, two cases reported adverse reactions, resulting in an incidence rate of 8.3%. Among them, 1 case had dizziness and headache (symptoms disappeared after 2 d of treatment, and the patient insisted on completing the light therapy), and 1 case had slight blurred vision (symptoms disappeared after 1 d of treatment, and insisted on completing the light therapy). One case in the control group experienced adverse reactions, and the incidence of adverse reactions was 4.2%. Headache appeared (symptoms disappeared without treatment, adhering to the completion of light therapy), all adverse reactions were not given special treatment, and they relieved themselves within 2 d. The difference in the rates of adverse reactions between the groups was not statistically significant (*p* = 1.00).

### 3.3 Changes in key outcome indicators

A linear mixed model was used to analyze the changes in blood glucose, lipid metabolism indicators, and cortisol rhythm parameters. The results showed that in the BLT group, FBG, cortisol amplitude and mesor were significantly lower than those at baseline and in the control group after treatment (*p* < 0.05). Additionally, the HAMD scores of patients in the BLT group after intervention were also significantly lower, but no significant differences were observed in the other metabolic indicators within or between the groups (see **Table 2**).

**Table 2:** Linear mixed model results for metabolic, cortisol, and clinical outcomes.

Outcome variable	Fixed effect	Estimate (β)	95% CI	*P*
FBG (mmol/L)	Group	-0.440	-0.600, -0.280	<0.001
	Time	0.351	0.257, 0.445	<0.001
	Group × Time	0.440	0.280, 0.600	<0.001
TC (mmol/L)	Group	-0.124	-0.264, 0.016	0.083
	Time	-0.124	-0.209, -0.039	0.004
	Group × Time	0.124	-0.016, 0.264	0.083
HDL-C (mmol/L)	Group	0.060	-0.014, 0.135	0.111
	Time	0.081	0.015, 0.148	0.018
	Group × Time	-0.069	-0.171, 0.032	0.176
TG (mmol/L)	Group	0.310	-0.037, 0.658	0.079
	Time	-0.180	-0.465, 0.105	0.211
	Group × Time	-0.306	-0.747, 0.135	0.170
Cortisol Mesor (ng/mL)	Group	1.039	0.083, 1.994	0.034
	Time	1.650	0.753, 2.547	0.001
	Group × Time	-1.370	-2.677, -0.064	0.040
Cortisol Amplitude (ng/mL)	Group	1.270	0.672, 1.868	<0.001
	Time	1.657	1.266, 2.049	<0.001
	Group × Time	-1.270	-1.869, -0.672	<0.001
Cortisol Acrophase (hours)	Group	0.472	-0.108, 1.051	0.110
	Time	0.001	-0.363, 0.365	0.995
	Group × Time	-0.472	-1.051, 0.108	0.111
Cortisol Period (hours)	Group	-2.196	-4.538, 0.146	0.066
	Time	-0.881	-2.428, 0.665	0.264
	Group × Time	2.193	-0.193, 4.579	0.072
HAMD Score	Group	9.301	4.317, 14.285	<0.001
	Time	21.689	17.074, 26.303	<0.001
	Group × Time	-9.549	-16.261, -2.836	0.006
HAMA Score	Group	5.208	1.340, 9.075	0.009
	Time	12.216	8.928, 15.503	<0.001
	Group × Time	-4.485	-9.328, 0.358	0.069

FBG, fasting blood glucose; TC, total cholesterol; HDL-C, high-density lipoprotein cholesterol; TG, triglycerides; HAMD, Hamilton Depression Scale; HAMA, Hamilton Anxiety Scale.

Bold values indicate statistically significant Group × Time interactions (*p* < 0.05).

After further controlling for gender factors, the results of the mixed model still supported the above findings (see **Supplementary Table 1**). In the BLT group, there was still a significant group × time interaction effect for FBG, cortisol mesor, and amplitude (*p* < 0.05).

### 3.4 Association between posttreatment changes in fasting blood glucose and cortisol rhythm parameters within the bright light therapy group

After controlling for age, gender, disease duration and drug dosage, the multiple linear regression analysis showed that the change in fasting blood glucose in the BLT group after treatment was significantly positively correlated with the change in cortisol mesor (β = 0.053, 95% CI: 0.016 to 0.122, *p* = 0.036), but there was no significant correlation with the changes in amplitude, acrophase and period (see **Table 3**).

**Table 3:** Association between posttreatment changes in fasting blood glucose and cortisol rhythm parameters within the bright light therapy group.

Cortisol parameters difference	FBG difference		
	Regression coefficient (β)	95%CI	*P*
Amplitude	0.051	-0.073,0.176	0.133
Acrophase	0.038	-0.181,0.257	0.188
Mesor	0.053	0.016,0.122	**0.036**
Period	0.034	-0.024,0.093	0.089

Model: Adjusted for age, gender, duration of disease, dose. Bold values indicate statistically significant (*p* < 0.05).

### 3.5 Association between posttreatment changes in cortisol rhythm parameters and Depression and Anxiety Symptoms within the two groups

The Pearson correlation analysis showed that in the BLT group, the changes in HAMD scores were significantly positively correlated with the changes in cortisol mesor and amplitude (r = 0.643, *p* = 0.018; r = 0.773, *p* = 0.003), but not with the changes in period and acrophase. In the control group, no significant correlation was observed between the cortisol rhythm parameters and the changes in HAMD or HAMA scores (see **Table 4**).

**Table 4:** Association between posttreatment changes in cortisol rhythm parameters and Depression and Anxiety Symptoms within the two group.

Group	Dependent variable	Mesor		Amplitude		Period		Acrophase	
		Pearson’s	*P*	Pearson’s	*P*	Pearson’s	*P*	Pearson’s	*P*
BLT	HAMD scores	0.643	**0.018**	0.773	**0.003**	-0.042	0.897	0.173	0.590
	HAMA scores	-0.302	0.316	-0.512	0.089	-0.067	0.835	-0.153	0.635
Control	HAMD scores	0.277	0.438	0.057	0.876	0.417	0.230	0.190	0.599
	HAMA scores	-0.186	0.607	0.072	0.843	-0.228	0.560	-0.482	0.158

Bold values indicate statistically significant (*p* < 0.05).

### 3.6 Association between posttreatment changes in fasting blood glucose and depression symptoms within the two groups

After controlling for the gender factor, the partial correlation analysis showed that the change in HAMD score in the BLT group was moderately positively correlated with the change in fasting blood glucose (β = 0.531, 95% CI: 0.138 to 0.848, *p* = 0.042), while there was no significant correlation in the control group (see **Table 5**).

**Table 5:** Association between posttreatment changes in FBG and Depression Symptoms within the two groups.

Group	Variable	HAMD Scores difference		
		regression coefficient (β)	95%CI	*P*
BLT	FBG difference	0.531	0.138, 0.848	**0.042**
Control	FBG difference	-0.116	-0.616,0.863	0.766

Model: Adjusted for gender.Bold values indicate statistically significant (*p* < 0.05).

### 3.7 Gender stratification sensitivity analysis

To further explore the influence of gender on the therapeutic effect, we conducted a gender-stratified sensitivity analysis (see **Supplementary Table 2**). The results showed that in the female subgroup, the light therapy had a more significant improvement trend on fasting blood glucose (β = -1.346, *p* = 0.065), but it did not reach statistical significance; in the male subgroup, the above interaction effect was weaker.

## 4 Discussion

This study examined the impact of bright light therapy on circadian rhythm synchronization and metabolic health (glucose/lipid metabolism) in depressed individuals through a randomized controlled design. Conducted at a psychiatric specialist hospital, the study specifically enrolled hospitalized individuals with depressive disorders. Consequently, the findings are primarily applicable to the patient population receiving specialized psychiatric inpatient care.

The application of bright light therapy exerted a beneficial effect on glycemic control, manifested as lower fasting blood glucose in the depressed cohort, suggesting its potential to serve as an effective non-pharmacological approach for glycemic management. A tendency toward improved glucose regulation was noted in the BLT group compared to the control condition, lending support to prior findings reported by Nieuwenhuis and colleagues (27). A regimen of 10,000 lux light therapy, delivered in 30-minute increments over 10 sessions (totaling 10 hours), resulted in an immediate decline in blood glucose concentrations. The present investigation confirms that bright light intervention induces favorable alterations in glucose metabolism among subjects with depression. This finding is consistent with a growing consensus suggesting that modulating circadian rhythms is integral to metabolic health (33). In the control group, there was no improvement in blood glucose levels. This could be attributed to the fact that the exposure to low - intensity light in the control group was insufficient to drive meaningful circadian rhythm synchronization or metabolic regulation. High-intensity light, on the other hand, is a potent clock regulator that can acutely influence cortisol rhythms, thereby more effectively resetting SCN and downstream metabolic pathways. Research has indicated that high-intensity light therapy is more effective than low-intensity light therapy (34). However, interpretation of these results requires careful discussion of the potential confounding effects of antidepressant treatment. Since the effects of multiple psychotropic drugs on glucose metabolism are well documented, they are heterogeneous. The literature presents conflicting results on how antidepressants influence insulin sensitivity and glucose homeostasis, with divergent findings ranging from an elevated diabetes risk to neutral or even beneficial effects. It has also been found that the use of selective serotonin reuptake inhibitors (SSRIs) may improve glucose metabolism, whereas certain norepinephrine drugs may worsen glucose metabolism (35). During the initial weeks of therapy, SSRIs like sertraline and citalopram can enhance insulin sensitivity. Research suggests this transient benefit may result from serotonin’s role in regulating hepatic glucose output (36). Notably, the timing of metabolic assessment becomes critical. Because longitudinal studies have shown that long-term SSRI use (>6 months) may paradoxically increase diabetes risk through a cumulative effect on obesity (37). In summary, although our findings support bright light therapy as a promising adjunct to metabolic management of depression, pharmacological context remains an important moderator of effect. Clinical protocols should incorporate antidepressant pharmacogenomic analysis and continuous glucose monitoring to optimize personalized treatment strategies.

The current investigation failed to detect significant alterations in lipid metabolism, which may be attributed to the relatively short duration and moderate intensity of the light therapy regimen. This is consistent with a previous trial employing light therapy at 4000 lux for 5 hours per day, which reported elevated fasting triglyceride levels under bright light therapy compared to dim light (38). Accordingly, the notable lack of significant alteration in lipid measures could be attributable to the intensity and duration of light administered, a conclusion that warrants verification in subsequent research.

At the same time, we also found that the mesor and amplitude of cortisol decreased before and after the light therapy. At present, other study did not measure the circadian rhythm of cortisol by noninvasive acquisition, including detailed analysis of parameters such as period, amplitude, mesor, and acrophase. In the present trial, we administered bright light therapy (10,000 lux) for 30 minutes daily between 9:00 and 10:00 a.m. over a two-week period, with salivary cortisol sampled at nine timepoints across two consecutive days. This study used a detailed parametric analysis to comprehensively assess the rhythmicity of patients with depression before and after light intervention. The present data support and extend the findings previously reported by Kostoglou-Athanassiou and colleagues (39), regarding the cortisol-lowering effect of bright light therapy. However, the existing literature presents a complex picture, with studies also reporting increased (40, 41) or negligible changes (42–46) in cortisol levels following light exposure. This discrepancy suggests that key parameters such as treatment duration, light intensity, and timing relative to biological rhythms are critical moderators of cortisol response (47). This protocol differs from several prior studies that used longer daily exposure durations (e.g., 2–5 hours), varied timing of light exposure (e.g., morning vs. evening), or shorter intervention periods (e.g., single or several days). Such methodological variations—particularly in exposure timing relative to the circadian phase and total cumulative light dose—likely contribute to the heterogeneity in reported cortisol outcomes.

Our study also found that fasting blood glucose and the cortisol mesor after light therapy showed a strong correlation. Currently, the majority of existing research has emphasized the isolated impact of light therapy on either fasting blood glucose or cortisol levels, with limited investigation into their combined effects. Although the study of Jung, C. M., et al. focused on cortisol, it was mentioned that changes in cortisol levels may indirectly affect fasting blood glucose by affecting insulin sensitivity (47). Therefore, our findings suggest that the improvement in fasting blood glucose observed following bright light therapy is associated with changes in cortisol rhythmicity. Further experimental research is required to elucidate the underlying mechanisms through which cortisol influences insulin sensitivity. Additionally, the potential therapeutic efficacy of bright light therapy in managing dysglycemia among depressed individuals warrants investigation.

We further investigated the effect of bright light therapy on depressive symptoms. After the intervention with bright light, depressive symptoms were significantly alleviated, and this result was confirmed by Menegaz de Almeida’s previous retrospective study (48). Their systematic review included a meta-analysis of 11 randomized clinical trials, pooling data from over 850 participants. The study found that among the population using bright light therapy (at least 30 minutes per day, 10,000 lux of white light), the remission rate of non-seasonal depression was 41%. However, the improvement of anxiety symptoms was not obvious with light therapy, possibly because anxiety symptoms are closely related to sleep quality. More and more studies have shown that phototherapy can significantly improve the sleep quality of participants and alleviate symptoms related to anxiety (49, 50).

This investigation revealed that, following gender adjustment, higher HAMD scores were predictive of increased fasting blood glucose levels solely within the BLT group, a relationship not identified in the control group. This finding indicates that the observed association between light therapy and changes in fasting blood glucose may be related to alterations in cortisol regulation. This correlation was not present in controls, implying that the interaction between blood glucose and depressive symptoms may be state-dependent and require specific physiological conditions, such as bright light-induced hormonal changes, to manifest.

In the process of light therapy, a few patients experienced discomfort, such as dizziness and headache, but the symptoms were mild and transient and improved spontaneously without treatment. The overall adverse effect profile was favorable, in agreement with previously published data (51). During the light therapy, the patient did not have manic episodes and other adverse reactions.

The patients recruited in this study were all inpatients, and diet and exercise were strictly controlled. Thus, the influence of these factors on fasting blood glucose levels was avoided, which could affect the accuracy of the study results. Our study has several limitations. To begin with, the proportion of men was higher in the BLT group than in the control group after randomization (58% vs. 29%), and residual confounding is possible, although the multivariable regression model was adjusted for sex. Moreover, the sample was dominated by women (56%), which was inconsistent with the gender distribution of the general population and may affect the external validity of the results. However, some studies have also indicated that women are more sensitive to the impact of bright light on the circadian rhythm system, while the responses at lower light levels do not exhibit significant gender differences (52). And, the generalizability of these results is restricted by the single-center origin and modest number of participants. Enhancing the wider relevance of future findings will therefore require studies that are both multi-institutional and incorporate a more balanced sex representation. In the future, we can expand the sample size, strictly control the confounding factors, and explore the effects of different light parameters (such as light intensity, wavelength, irradiation time, etc.) on fasting blood glucose optimization. In addition, the exclusion of patients with other primary psychiatric conditions, while necessary to ensure internal validity and isolate the effects of bright light therapy on depression and metabolic parameters, may limit the generalizability of our findings to the broader population of depressed individuals with comorbid psychiatric disorders. Additionally, although we excluded current smokers, smoking history was included in our statistical models to account for potential long-term metabolic influences. Finally, the duration of this trial was limited; extended observation and additional studies are necessary to assess the long-term efficacy and safety profile of high-intensity light therapy in regulating blood glucose among depressed individuals.

## 5 Conclusion

This study demonstrates that bright light therapy can improve fasting glucose levels in individuals with depression, likely mediated through modulation of cortisol rhythmicity. These findings offer preliminary evidence supporting the therapeutic potential of BLT for glycemic management in depressed inpatients. Further validation through larger, multi-center trials—including outpatient populations and patients with confirmed metabolic syndrome—is warranted to establish its broader applicability.

## Data Availability

The raw data supporting the conclusions of this article will be made available by the authors, without undue reservation.
