# Supplementary material for: The effect of bright light therapy on glycemic control and cortisol rhythmicity in depression: a randomized controlled trial
Source: Front Psychiatry. 2026 Feb 11;17:1743465. doi: 10.3389/fpsyt.2026.1743465 (PMC12932458; doi:10.3389/fpsyt.2026.1743465)
Supplement: Supplementary file 1 [file Table1.docx]

| **Supplementary Table 1. Linear mixed model results for metabolic, cortisol, and clinical outcomes (adjusted for sex)** | | | | |
| --- | --- | --- | --- | --- |
| Outcome Variable | Fixed Effect | Estimate (β) | 95% CI | *p* |
| **FBG (mmol/L)** | Group | -0.440 | -0.599, -0.280 | **<0.001** |
|  | Time | 0.351 | 0.257, 0.445 | **<0.001** |
|  | Group × Time | 0.440 | 0.280, 0.600 | ****<0.001**** |
| **TC (mmol/L)** | Group | -0.111 | -0.325,0.102 | 0.307 |
|  | Time | -0.122 | -0.255,0.012 | 0.073 |
|  | Group × Time | 0.112 | -0.103,0.327 | 0.306 |
| **HDL-C (mmol/L)** | Group | 0.053 | -0.024,0.130 | 0.171 |
|  | Time | 0.081 | 0.020, 0.142 | **0.010** |
|  | Group × Time | -0.068 | -0.163, 0.026 | 0.156 |
| **TG (mmol/L)** | Group | 0.376 | 0.026, 0.727 | ****0.035**** |
|  | Time | -0.181 | -0.457, 0.095 | 0.195 |
|  | Group × Time | -0.312 | -0.741, 0.116 | 0.151 |
| **Cortisol Mesor (ng/mL)** | Group | 1.171 | 0.186, 2.156 | **0.020** |
|  | Time | 1.689 | 0.887, 2.491 | **<0.001** |
|  | Group × Time | -1.353 | -2.548, -0.158 | ****0.027**** |
| **Cortisol Amplitude (ng/mL)** | Group | 1.086 | 0.142, 2.030 | **0.025** |
|  | Time | 1.631 | 0.866, 2.396 | **<0.001** |
|  | Group × Time | -1.209 | -2.352, -0.065 | ****0.039**** |
| **Cortisol Acrophase (hours)** | Group | 0.321 | -0.610, 1.252 | 0.494 |
|  | Time | 0.011 | -0.745, 0.767 | 0.978 |
|  | Group × Time | -0.522 | -1.671, 0.626 | 0.367 |
| **Cortisol Period (hours)** | Group | -2.199 | -4.159, -0.239 | **0.028** |
|  | Time | -0.868 | -2.458, 0.722 | 0.280 |
|  | Group × Time | 2.221 | -0.191, 4.634 | 0.071 |
| **HAMD Score** | **Group** | **9.302** | **4.212, 14.392** | **0.001** |
|  | **Time** | **21.867** | **17.604,26.130** | **<0.001** |
|  | **Group × Time** | **-9.782** | **-16.061,-3.504** | ****0.003**** |
| **HAMA Score** | **Group** | **5.169** | **1.257, 9.080** | **0.010** |
|  | **Time** | **12.211** | **8.924, 15.499** | **<0.001** |
|  | **Group × Time** | **-4.484** | **-9.327, 0.358** | **0.069** |
| ****Abbreviations:**** FBG, fasting blood glucose; TC, total cholesterol; HDL-C, high-density lipoprotein cholesterol; TG, triglycerides; HAMD, Hamilton Depression Scale; HAMA, Hamilton Anxiety Scale. ****Notes:**** Bold values indicate statistically significant Group × Time interactions ( *p* < 0.05) | | | | |

| **Supplementary Table 2. Sex-stratified sensitivity analyses of Group × Time interaction effects** | | | |
| --- | --- | --- | --- |
| Outcome | Gender | β (95% CI) | *p* |
| **FBG (mmol/L)** | Female | **-0.614** (-1.407, 0.180) | 0.128 |
|  | Male | -0.077 (-0.814, 0.660) | 0.836 |
| **Cortisol Mesor (ng/mL)** | Female | **-1.346** (-2.778, 0.087) | **0.065** |
|  | Male | **-2.392** (-5.229, 0.445) | 0.094 |
| **Cortisol Amplitude (ng/mL)** | Female | -1.100 (-3.174, 0.973) | 0.279 |
|  | Male | -2.065 (-5.052, 0.923) | 0.153 |
| ****Abbreviations:**** FBG, fasting blood glucose;  ****Notes:**** β represents the Group × Time interaction estimate. Negative β values indicate greater reduction in the BLT group compared to control. | | | |
